# Awareness and Use of Heated Tobacco Products among Youth Smokers in Hong Kong: A Cross-Sectional Study

**DOI:** 10.3390/ijerph17228575

**Published:** 2020-11-19

**Authors:** Laurie Long Kwan Ho, William Ho Cheung Li, Ankie Tan Cheung, Wei Xia, Tai Hing Lam

**Affiliations:** 1 School of Nursing, University of Hong Kong, Hong Kong, China; longkwan@hku.hk (L.L.K.H.); tankie@hku.hk (A.T.C.); xiavive@hku.hk (W.X.); 2 School of Public Health, University of Hong Kong, Hong Kong, China; hrmrlth@hku.hk

**Keywords:** heated tobacco products, awareness, tobacco, smoking, substance use, youth

## Abstract

The growing popularity of heated tobacco products (HTPs) among youth may act as a gateway for smoking and jeopardize youth health. We aimed to describe the use of HTPs among youth smokers in Hong Kong and examine their risk awareness of HTPs as well as awareness of the proposed legislation. We conducted retrospective data analyses on the Youth Quitline Cohort (*n* = 731). We extracted participants’ sociodemographic data, smoking profiles, and HTP use from 1 January 2017. Participants’ HTP use increased from 5.7% in 2017 to 37.9% in 2020. Among the 731 participants, 175 were HTP users and 556 were HTP nonusers. Compared with nonusers, a significantly higher proportion of HTP users had tried using other tobacco products at least once. The most common reason for using HTPs was curiosity. HTP users were more likely than nonusers to misclassify HTPs as e-cigarettes; agree that HTPs were healthier and contained fewer harmful substances than conventional cigarettes; consider HTPs as a smoking cessation aid; and believe that HTPs could reduce conventional cigarette consumption. Overall, 61.2% of youth smokers disagreed with banning HTPs. Risk awareness of HTPs among youth might affect their likelihood of using these products. Stricter regulations on advertising and intensive health education are imperative to avoid misleading information and limit youth exposure to such harmful products.

## 1. Introduction

The increasing use of alternative tobacco products among youth has offset the overall decrease in conventional cigarette use and become a global public health concern [1,2]. Spurred by increasing evidence unmasking the detrimental health effects of e-cigarettes, including undermining abstinence [3], encouraging dual use with increased toxicant exposures [4,5], and acting as a gateway for youth smoking [6], many countries and regions have enacted legislation regulating or completely banning such products [7,8]. Unlike e-cigarettes, heated tobacco products (HTPs) have recently been revived by major product innovations and product repositioning to tap into growing markets [9]. Despite the fact that the health effects remain controversial, HTPs have been aggressively promoted and marketed as a safer alternative to conventional cigarettes because they generate a nicotine containing aerosol by heating tobacco at a lower temperature than combusted cigarettes [10]. In July 2020, the US Food and Drug Administration (FDA) authorized the marketing of the I-Quit-Ordinary-Smoking (IQOS) tobacco heating system, which is used in HTPs from a leading manufacturer, and recognized these products as modified risk tobacco products [11]. Although IQOS was granted an exposure modification order by the FDA, the FDA reiterated that these products were neither safe nor “FDA approved.” However, the effect of this FDA authorization on the use of IQOS among youth is unpredictable. 

The potential risks of introducing HTPs into the market should not be underestimated because it may increase youth exposure and promote tobacco use among non-smokers, especially young people [12]. A dramatic increase in HTP use among youth has been observed in countries that have authorized the marketing of such products, which suggests that the trendy packaging and various flavors of HTPs mostly cater for young people [1,13]. A longitudinal study showed that there was a threefold increase in HTP use among youth aged 15–19 years in Japan, from 0.6% in 2015 to 2.0% in 2017 [14]. A study from South Korea also found that 2.8% of youth aged 12–18 years had used HTPs after 1 year of these products being legally available in the market [15]. Despite the sale of HTPs being prohibited in Hong Kong, 2.3% of youth reported that they had used such products [16]. In comparison with the findings from Japan and South Korea, HTP use among youth in Hong Kong warrants attention. As the most Westernized city in China, Hong Kong has the lowest smoking prevalence (10.2%) in the developed world and a low youth smoking prevalence (2.5%) [16,17]. Despite this low youth smoking prevalence, growing evidence shows that two-thirds of smokers died prematurely, especially those who started smoking at a young age [18,19,20]. Youth smokers are also more likely to continue smoking in adulthood [21]. To prevent alternative tobacco products from undermining tobacco control efforts and jeopardizing youth health, the Hong Kong government has proposed legislative amendments to enact a total ban on such products. However, these amendments have failed to pass because of filibustering by some legislators and disruption of Bills Committee meetings by the COVID-19 pandemic. Those legislators supported banning e-cigarettes, but proposed allowing the sale of HTPs by classifying them as a safer alternative to conventional cigarettes. 

Despite the absence of compelling evidence regarding the harmful effects of HTPs, increasing evidence indicates that the IQOS tobacco heating system may not be as safe as claimed by the manufacturer; this is because of its potentially harmful constituents and the increased concentration of nicotine and other harmful chemicals emitted by HTP devices [22,23,24]. Given that all tobacco products are harmful [25], it is of paramount importance to prevent the initiation and decrease the prevalence of alternative tobacco product use among youth. Previous studies have showed that the perceived risks were associated with the likelihood and pattern of tobacco use [26,27,28,29]. By understanding risk awareness of HTPs among youth, healthcare professionals could predict their smoking behavior [30,31,32], which may facilitate the design of appropriate interventions for promoting smoking cessation among youth and preventing youth smoking. Most available studies examining awareness and use of HTPs among young adults or adults have observed high levels of awareness and increasing use [33,34,35,36], whereas data regarding attitudes toward and use of HTPs among youth remain scarce. To fill this gap in the literature and inform health initiatives and regulatory decision-making, the present study aimed to (1) examine youth smokers’ risk awareness of HTPs; (2) determine youth smokers’ awareness of the proposed legislation; and (3) describe HTP use among youth smokers in Hong Kong. 

## 2. Materials and Methods

### 2.1. Study Design and Participants

We conducted retrospective data analyses on the Youth Quitline (YQ) Cohort of youth smokers. Details of the YQ service have been described elsewhere [37,38]. YQ participants were: (1) aged 14–25 years, (2) current smokers who had consumed one or more tobacco product types in the previous 30 days, (3) Cantonese speakers, and (4) willing to receive telephone counseling to support smoking cessation. Youth smokers who had communication barriers or had participated in other smoking cessation programs or services were excluded. 

YQ has recorded the use of HTPs among youth smokers since 1 January 2017. In this study, participants were classified as HTP users or nonusers. Individuals who had used at least one HTP in the previous 30 days were defined as HTP users. We further classified HTP users as exclusive HTP users or dual users (those who had used HTPs with other tobacco products in the previous 30 days). Youth smokers who had used tobacco products except HTPs in the previous 30 days were classified as HTP nonusers. The operational definition of smokers had been used in previous smoking research among youth [38]. 

### 2.2. Methods

We extracted participants’ sociodemographic data, including sex, age, marital status, educational attainment, and employment status. We also collected participants’ smoking profiles, which included age at starting smoking, daily cigarette consumption, previous use of other tobacco products (e.g., e-cigarettes) except HTPs, previous quit attempts, and readiness to quit. Participants’ nicotine dependency was assessed by the Fagerström Test for Nicotine Dependency, which is a valid and reliable tool for assessing nicotine addiction [39]. We extracted data for participants’ risk awareness of HTPs and their awareness of the proposed legislation, which were available from 1 January 2018. We asked participants to respond to the statements regarding their risk awareness of HTPs as well as awareness of the proposed legislation, for instance, “Do you agree that HTPs such as IQOS is a type of e-cigarette or equivalent to e-cigarettes?” For HTP users, we also examined their reasons for using such products. 

All telephone conversations were audio-recorded with participants’ permission. Verbal informed consent was obtained from youth smokers prior to joining this study. We assured youth smokers about the voluntary nature of participation, in which they could withdraw from the study at any time, and that the confidentiality of the information collected would be maintained. 

### 2.3. Statistical Analysis

Statistical analyses were conducted using SPSS version 25.0 for Windows (IBM Corp., Armonk, NY, USA). Use of HTPs and reasons for use were described as percentages. Sociodemographic characteristics and smoking profiles were compared between HTP users and nonusers using χ^2^ tests for categorical variables and independent sample *t*-tests for continuous variables. We also used χ^2^ tests to compare risk awareness of HTPs as well as awareness of the proposed legislation between HTP users and nonusers. A *p*-value < 0.05 was considered to be statistically significant.

### 2.4. Ethical Approval

Ethical approval for this study was obtained from the Institutional Review Board of the University of Hong Kong/Hospital Authority Hong Kong West Cluster (UW 05-185 T/848) in 2020. 

## 3. Results

**Figure 1:**
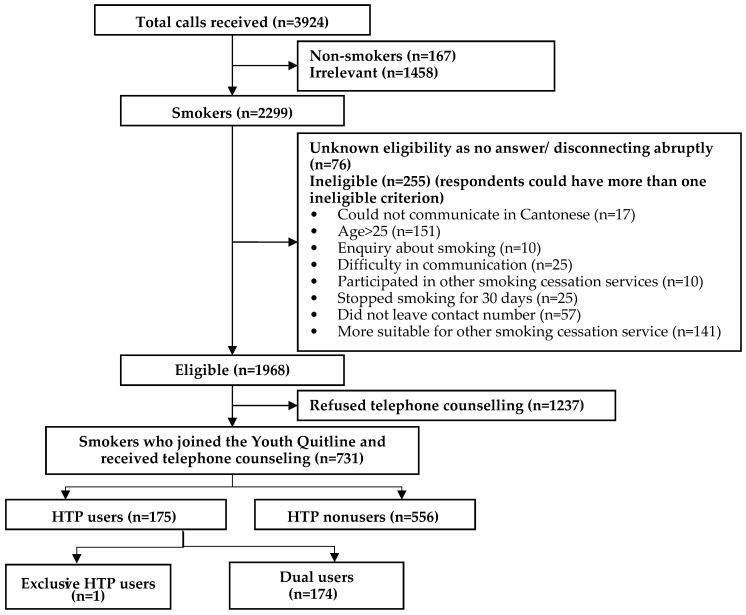
Recruitment of the Youth Quitline from 1 January 2017 to 30 June 2020.

**Figure 2:**
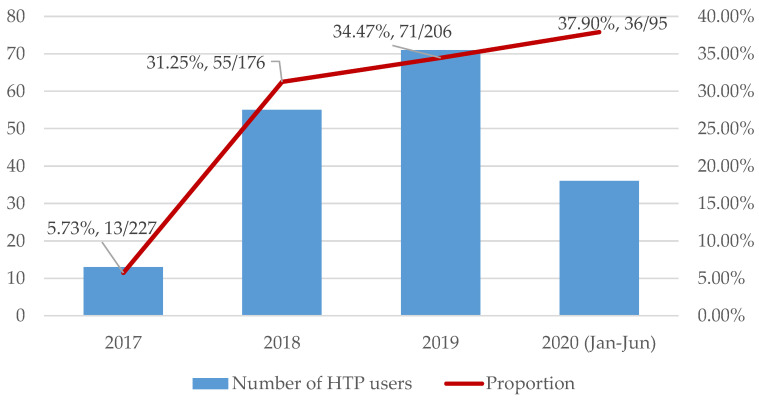
Usage of heated tobacco products (HTPs) in smokers from Youth Quitline 1 January 2017 to 30 June 2020.

From 1 January 2017 to 30 June 2020, we received 3924 incoming calls, of which 1968 were eligible for the YQ service (Figure 1). Of these, 731 youth smokers agreed to receive telephone counseling to support smoking cessation; 175 (23.9%) were HTP users and 556 (76.1%) were HTP nonusers. Since 1 January 2018, 477 participants have completed telephone follow-up and were asked about their risk awareness of HTPs. Among HTP users, one participant was an exclusive HTP user and 174 were dual users. Figure 2 shows HTP use increased from 5.73% in 2017 to 37.90% in 2020.

HTP users and nonusers had similar sociodemographic characteristics and smoking profiles, with the exception of age and employment status (Table 1). HTP users had a significantly higher mean age than nonusers (19.7 years vs. 19.1 years, *p* = 0.02, d = 0.20). A significantly higher proportion of HTP users than nonusers were part-time or full-time employees (73.1% vs. 55.1%, *p* = 0.01, V = 0.12). Otherwise, most participants were male (83.0%), single (98.3%), had an upper secondary educational level (50.1%), and had a mild level of nicotine dependency (68.4%). 

Compared with HTP nonusers, a significantly higher proportion of HTP users had tried using other tobacco products at least once (87.4% vs. 66.7%, *p* < 0.001, φ = 0.21). No significant differences were found between HTP users and nonusers in age at starting smoking (14.7 years vs. 14.6 years, *p* = 0.59), daily cigarette consumption (8.7 vs. 9.3, *p* = 0.32), previous quit attempts (76.6% vs. 72.3%, *p* = 0.29), and readiness to quit (*p* = 0.36).

Table 2 shows participants’ risk awareness of HTPs and their awareness of the proposed legislation. Compared with nonusers, HTP users were more likely to misclassify HTPs as e-cigarettes (36.4% vs. 25.3%, *p* = 0.01, V = 0.13) and to agree that HTPs were healthier (38.9% vs. 19.9%, *p* < 0.001, V = 0.21) and contained fewer harmful substances (38.9% vs. 20.5%, *p* < 0.001, V = 0.20) than conventional cigarettes. HTP users were also more likely that nonusers to consider HTPs as a smoking cessation aid (32.5% vs. 14.3%, *p* < 0.001, V = 0.22) and believe that HTPs could reduce conventional cigarette consumption (32.1% vs. 18.1%, *p* = 0.001, V = 0.16). Regardless of the use of HTPs, 61.2% of youth smokers disagreed with banning HTPs and 54.3% disagreed that banning alternative tobacco products would be a more efficient strategy to control the consumption of such products than promoting smoking cessation. However, no significant difference was found in awareness of the proposed legislation between HTP users and nonusers. 

Table 3 shows that participants’ main reasons for using HTPs were curiosity (46.7%), peer influence (33.3%), perceived health benefits (9.3%), and as a smoking cessation aid (8.7%).

## 4. Discussion

This study found that approximately one-fourth of YQ participants had used HTPs, and the number of youth using HTPs had increased by approximately sevenfold in the last 3 years. The growing use of HTPs among youth smokers shared similar usage pattern among other age groups (e.g., young adults or adults) [15,34,35,36]. This represents an emerging public health challenge that may warrant stricter measures on exposure and access to such harmful products among youth.

Our findings highlight a significant difference in risk awareness toward HTPs between users and nonusers. In general, HTP users were more likely than nonusers to believe that HTPs were less harmful to their health than conventional cigarettes, and could reduce the consumption of conventional cigarettes and help in quitting smoking. This implied that the perceived risk level of HTP use among youth might affect their likelihood of using such products, and thus suggested the need of further investigation. HTP manufacturers, such as Philip Morris International (PMI), have attempted to downplay the harmful effects of HTPs and market them as a safer alternative to conventional cigarettes. Although radio and television marketing of these products is banned, retail marketing and advertising on the Internet are easily accessed by youth. Extensive promotions also increase exposure to HTP advertisements among young people, resulting in higher odds of using such products [40,41]. The implementation of stringent regulations on tobacco advertising is imperative to reduce youth exposure to these products. With the FDA authorization, PMI can market the IQOS system as a tobacco product that “significantly reduces the production of harmful and potentially harmful chemicals” and “reduces body’s exposure to harmful or potentially harmful chemicals by switching completely from combusted cigarettes to IQOS” [11]. Misunderstanding of the message in advertisements among youth may disseminate false beliefs, and create erroneous impressions of the characteristics and health hazards of HTPs. Intensive health education and promotion should be targeted at youth population to reiterate and advocate that there is no safe tobacco product, aiming at preventing smoking initiation among youth. It is also crucial to avoid any misinformation in the media that may mislead youth into believing that the IQOS system is safe for use and approved by the FDA.

Notably, we found that smoking cessation was not reported as a main reason for HTP use among youth. Instead, more than half of the youth smokers had started to use such products because of curiosity, which implies that we cannot reject the gateway hypothesis of HTP use among youth. Our findings also showed that most HTP users were dual users, which might contradict the claims supported by the FDA that completely substituting HTPs for combusted cigarettes can reduce body exposure to harmful chemicals [11]. However, further research is needed for determining the level of youth exposure to HTPs. Furthermore, our findings indicate that youths who had used HTPs were older and employed. This might be because these participants could afford more expensive tobacco products such as HTPs. The present findings provide a reference for the target population in future smoking cessation promotions designed for youth, especially in health education pertinent to alternative tobacco products.

Reviewing the situation in Hong Kong, the government should revisit the proposed ban without exemption for HTPs in the new Legislative Council. However, our results show that a majority of youth smokers disagreed with banning HTPs. This might be because of misconceptions or false beliefs about the characteristics and health hazards of HTPs. The results suggest that rigorous community campaigns are needed to clarify the exposure modification orders issued by the FDA and provide more evidence to support the total ban and enhance the public’s understanding of the legislation. In particular, future studies should investigate the possible gateway effect of HTP use and estimate the level of youth exposure to such products.

Our study had some limitations. First, the generalizability of our findings might be limited as we only included YQ service users though YQ is the only youth-targeted smoking cessation hotline in Hong Kong. Second, potential confounders might have been underexplored by our use of a cross-sectional study design. Further studies may consider comparing the quit rate between HTP users and nonusers to build understanding of the impact of HTP use on smoking cessation among youth.

## 5. Conclusions

The results indicate a growing use and misconceptions about HTPs among youth smokers. This suggests an imperative need to further investigate the possible gateway effect of HTP use and estimate the level of youth exposure to such products in future studies with the aim of generating evidence to inform health initiatives and regulatory decision-making. Stricter regulations on advertising and intensive health education on the harmful effects of HTPs are of paramount importance to avoid misleading information and increase risk awareness of such products.

## Figures and Tables

**Table 1:** Sociodemographic characteristics and smoking profiles of the study sample.

Variable	No./Total No. (%) ^a^			
	All (*n* = 731)	HTP Nonusers (*n* = 556)	HTP Users (*n* = 175)	*p*-Value
Age, mean (SD), y	19.3 (2.9)	19.1 (2.9)	19.7 (3.0)	0.02
Sex				
Male	607/731 (83.0)	457/556 (82.2)	150/175 (85.7)	0.30
Female	124/731 (17.0)	99/556 (17.8)	25/175 (14.3)	
Marital Status				
Single	680/692 (98.3)	515/525 (98.1)	165/167 (98.8)	0.74
Married/cohabitated	12/692 (1.7)	10/525 (1.9)	2/167 (1.2)	
Educational attainment				
Lower secondary	89/700 (12.7)	74/533 (13.9)	15/167 (8.9)	0.12
Upper secondary	351/700 (50.1)	276/533 (51.8)	75/167 (45.0)	
Tertiary	260/700 (37.2)	183/533 (34.3)	77/167 (46.1)	
Employment status				
Full-time students	228/707 (32.3)	189/536 (35.3)	39/171 (22.8)	0.02
Full-time students and part-time employed	271/707 (38.3)	193/536 (36.0)	78/171 (45.6)	
Employed	182/707 (25.7)	135/536 (19.1)	47/171 (27.5)	
Unemployed	26/707 (3.7)	19/536 (2.7)	7/171 (4.1)	
Age at starting smoking, mean (SD), y	14.6 (2.9)	14.6 (2.8)	14.7 (3.2)	0.59
Daily cigarette consumption, mean (SD), No.	9.2 (7.5)	9.3 (7.8)	8.7 (6.5)	0.32
Previous use of other tobacco products (e.g., e-cigarettes) except HTPs				
Yes	524/731 (71.7)	371/556 (66.7)	153/175 (87.4)	<0.001
No	207/731 (28.3)	185/556 (33.3)	22/175 (12.6)	
Previous quit attempts (all tobacco products)				
Yes	536/731 (73.3)	402/556 (72.3)	134/175 (76.6)	0.29
No	195/731 (26.7)	154/556 (27.7)	41/175 (23.4)	
Readiness to quit (all tobacco products)				
Pre-contemplation	292/731 (39.9)	228/556 (41.0)	64/175 (36.6)	0.36
Contemplation	191/731 (26.1)	149/556 (26.8)	42/175 (24.0)	
Preparation	173/731 (23.7)	124/556 (22.3)	49/175 (28.0)	
Action	75/731 (10.3)	55/556 (9.9)	20/175 (11.4)	
Nicotine dependency by the Fagerström Test				
Mild, 0–3	492/719 (68.4)	369/548 (67.3)	123/171 (71.9)	0.52
Moderate, 4–5	163/719 (22.7)	128/548 (23.4)	35/171 (20.5)	
Severe, 6–10	64/719 (8.9)	51/548 (9.3)	13/171 (7.6)	

HTPs = heated tobacco products; e-cigarettes = electronic cigarettes; ^a^ sample sizes varied because of missing data on some variables.

**Table 2:** Youth smokers’ risk awareness of HTPs and their awareness of the proposed legislation.

	No./Total No. (%) ^a^			
	All (*n* = 477)	HTP Nonusers (*n* = 315)	HTP Users (*n* = 162)	*p*-Value
HTPs are e-cigarettes				
Agree/strongly agree	135/462 (29.2)	76/300 (25.3)	59/162 (36.4)	0.01
Disagree/strongly disagree	264/462 (57.1)	185/300 (61.7)	79/162 (48.8)	
Do not know	63/462 (13.6)	39/300 (13.0)	24/162 (14.8)	
HTPs are not addictive				
Agree/strongly agree	71/460 (15.4)	41/300 (13.7)	30/160 (18.8)	0.13
Disagree/strongly disagree	326/460 (70.9)	219/300 (73.0)	107/160 (66.9)	
Do not know	63/460 (13.7)	40/300 (13.3)	23/160 (14.4)	
HTPs are better than conventional cigarettes in terms of health				
Agree/strongly agree	125/474 (26.4)	62/312 (19.9)	63/162 (38.9)	<0.001
Disagree/strongly disagree	274/474 (57.8)	199/312 (63.8)	75/162 (46.3)	
Do not know	75/474 (15.8)	51/312 (16.3)	24/162 (14.8)	
HTPs contain less harmful substances than conventional cigarettes				
Agree/strongly agree	127/474 (26.8)	64/312 (20.5)	63/162 (38.9)	<0.001
Disagree/strongly disagree	272/474 (57.4)	197/312 (63.1)	75/162 (46.3)	
Do not know	75/474 (15.8)	51/312 (16.3)	24/162 (14.8)	
HTPs can help with smoking cessation				
Agree/strongly agree	92/451 (20.4)	43/300 (14.3)	49/151 (32.5)	<0.001
Disagree/strongly disagree	303/451 (67.2)	212/300 (70.7)	91/151 (60.2)	
Do not know	56/451 (12.4)	45/300 (15.0)	11/151 (7.3)	
HTPs can reduce conventional cigarette consumption				
Agree/strongly agree	106/461 (23.0)	54/299 (18.1)	52/162 (32.1)	0.001
Disagree/strongly disagree	287/461 (62.3)	200/299 (66.9)	87/162 (53.7)	
Do not know	68/461 (14.8)	45/299 (15.1)	23/162 (14.2)	
HTPs should be banned				
Agree/strongly agree	101/466 (21.7)	66/307 (21.5)	35/159 (22.0)	0.79
Disagree/strongly disagree	285/466 (61.2)	182/307 (59.3)	103/159 (64.8)	
Do not know/No comment	80/466 (17.2)	59/307 (19.2)	21/159 (13.2)	
Government should regulate HTPs but not ban				
Agree/strongly agree	228/462 (49.4)	140/300 (46.7)	88/162 (54.3)	0.12
Disagree/strongly disagree	168/462 (36.4)	116/300 (38.7)	52/162 (32.1)	
Do not know/No comment	66/462 (14.3)	44/300 (14.7)	22/162 (13.6)	
Banning of e-cigarettes and HTPs would be more efficient to control the consumptions of these products than promoting smoking cessation				
Agree/strongly agree	141/462 (30.5)	97/300 (32.3)	44/162 (27.2)	0.21
Disagree/strongly disagree	251/462 (54.3)	157/300 (52.3)	94/162 (58.0)	
Do not know/No comment	70/462 (15.2)	46/300 (15.3)	24/162 (14.8)	

^a^ Sample sizes varied because of missing data on some variables.

**Table 3:** Reasons for using HTPs.

Reason for Using HTPs ^b^	No./Total No. (%) ^a^
Curiosity	70/150 (46.7)
Peer influence	50/150 (33.3)
Perceived health benefits	14/150 (9.3)
Use as a smoking cessation aid	13/150 (8.7)
Others	3/150 (2.0)

^a^ Missing data were excluded; ^b^ only HTP users were asked the reason for using HTPs.
